# A tool for visualizing protein motions in time-resolved crystallography

**DOI:** 10.1063/1.5126921

**Published:** 2020-04-01

**Authors:** Cecilia Wickstrand, Gergely Katona, Takanori Nakane, Przemyslaw Nogly, Joerg Standfuss, Eriko Nango, Richard Neutze

**Affiliations:** 1 Department of Chemistry and Molecular Biology, University of Gothenburg, Box 462, SE-40530 Gothenburg, Sweden; 2 Department of Biological Sciences, Graduate School of Science, The University of Tokyo, Tokyo 113-0032, Japan; 3 MRC Laboratory of Molecular Biology, Cambridge CB2 0QH, United Kingdom; 4 Department of Biology, Institute of Molecular Biology and Biophysics, ETH Zurich, 8093 Zürich, Switzerland; 5 Laboratory of Biomolecular Research, Department of Biology and Chemistry, Paul Scherrer Institute, 5232 Villigen PSI, Switzerland; 6 Department of Cell Biology, Graduate School of Medicine, Kyoto University, Yoshidakonoe-cho, Sakyo-ku, Kyoto 606-8501, Japan; 7 RIKEN SPring-8 Center, 1-1-1, Kouto, Sayo-cho, Sayo-gun, Hyogo 679-5148, Japan

## Abstract

Time-resolved serial femtosecond crystallography (TR-SFX) at an x-ray free electron laser enables protein structural changes to be imaged on time-scales from femtoseconds to seconds. It can, however, be difficult to grasp the nature and timescale of global protein motions when structural changes are not isolated near a single active site. New tools are, therefore, needed to represent the global nature of electron density changes and their correlation with modeled protein structural changes. Here, we use TR-SFX data from bacteriorhodopsin to develop and validate a method for quantifying time-dependent electron density changes and correlating them throughout the protein. We define a spherical volume of difference electron density about selected atoms, average separately the positive and negative electron difference densities within each volume, and walk this spherical volume through all atoms within the protein. By correlating the resulting difference electron density amplitudes with time, our approach facilitates an initial assessment of the number and timescale of structural intermediates and highlights quake-like motions on the sub-picosecond timescale. This tool also allows structural models to be compared with experimental data using theoretical difference electron density changes calculated from refined resting and photo-activated structures.

## INTRODUCTION

Structural dynamics are as intrinsic to the protein function as the protein structure. All enzymes undergo conformational changes at their active site and often throughout the protein. Understanding how evolution has optimized both the structure and structural dynamics to achieve specific functions is a major goal of life-science. Time-resolved crystallography has been developed over several decades with the goal of providing quantitative data on how structural dynamics guide the reaction pathway of enzyme catalyzed reactions.^1^ Synchrotron based time-resolved Laue-diffraction has been applied to study light induced co-factor isomerization in photoactive yellow protein (PYP).^2–4^ Photo-dissociation of carbon monoxide from the heme group of myoglobin,^5,6^ dimeric hemoglobin,^7^ and an isolated domain of the oxygen sensor FixL^8^ has also been heavily studied using this method. Time-resolved Laue diffraction studies of a photosynthetic membrane protein^9^ as well as other chemically driven reactions^10,11^ have also revealed structural changes. A rapid period of growth of the field of time-resolved protein crystallography was sparked by the advent of time-resolved serial femtosecond crystallography (TR-SFX) at an x-ray free electron laser (XFEL). Both the extreme peak brilliance and the femtosecond timescale of the x-ray pulses generated at XFELs have been exploited to focus approximately 10^11^ photons through protein microcrystals a few micrometers in size.^12–15^ Exposure to very intense x-ray pulses led to the rapid explosion of these microcrystal samples, but the principle of diffraction-before-destruction^16^ ensures that x-ray diffraction data are scattered toward the detector before the sample is destroyed.

Serial Femtosecond Crystallography (SFX) has become a powerful crystallographic method because it averages diffraction patterns recorded from thousands of microcrystals, and therefore, errors in individual measurements tend to wash out. An important demonstration of the power of this method was the first TR-SFX study of light induced structural changes in PYP,^17^ which was benchmarked against earlier Laue diffraction studies using synchrotron radiation. This stimulated a rapid growth in the number of proteins studied by time-resolved diffraction including sub-picosecond structural dynamics of PYP,^18^ ultrafast changes in myoglobin following the photo-dissociation of bound carbon monoxide,^19^ structural changes in photo-switchable fluorescent proteins,^20^ new insights into the reduction of oxygen to water in the oxygen evolving center of photosystem II,^21–23^ and the evolution of structural changes within the light-driven proton pump bacteriorhodopsin (bR).^24,25^ Reaction triggering approaches for time-resolved diffraction studies are also progressing beyond the study of naturally light driven proteins with the development of microjet technologies that facilitate rapid mixing of crystals with chemical reagents,^26,27^ the use of caged-compounds in TR-SFX studies,^28^ and the observation of structural changes in proteins upon their exposure to pulsed electric fields^29^ or THz radiation.^30^ Conformational perturbations have also been characterized as a consequence of changes in temperature using multi-temperature crystallography,^31^ and rapid temperature-jumps have induced protein conformational changes that can be visualized using time-resolved x-ray scattering.^32^

As the field grows, there is a corresponding need to develop tools to quantify and characterize structural motions within proteins. This is particularly difficult for ultrafast motions for which the concept of a protein-quake (or ultrafast correlated motions) on the sub-picosecond to picosecond timescale is becoming established^19,33–36^ and for the excitation of motions using electric fields or non-ionizing radiation.^29,30^ In these cases, the time-honored method of an experienced crystallographer making subjective assessments of structurally meaningful movements around a chromophore or cofactor and building up a structural interpretation from that starting point may not be sufficient. Three approaches to this challenge have been developed including singular value decomposition (SVD) of a sequence of difference Fourier electron density maps,^8,37^ a linear decomposition of a matrix of time-dependent difference electron density amplitudes associated with selected residues,^24^ or a cluster-based approach that identifies voxels for which the time dependence of the difference electron density is most similar.^38^

Here, we develop another approach for representing difference electron density changes from a sequence of time-resolved diffraction studies by first reducing the presentation of three-dimensional difference electron density changes to one-dimensional plots. The underlying principle of this approach is similar to how we presented a time-dependent sequence of difference electron density changes for selected residues in earlier publications^24,25^ but removes subjective choices as to which difference electron density peak corresponds to which residue, avoids issues associated with the location of a selected difference electron density peak varying from one map to the next, avoids the need for manually reading peak maxima and minima from difference electron density maps, and is easily applied to any region of the protein, or the protein as a whole, as required. We illustrate the power of this representation using difference Fourier electron density maps and the results of structural refinement from two recent TR-SFX studies of bacteriorhodopsin.^24,25^ This reanalysis of time-dependent electron density changes does not touch upon an important but separate scientific question concerning the possible impact of multi-photon excitation of the retinal chromophore on the protein's observed trajectory.^39^ We first demonstrate this method using TR-SFX data recorded for thirteen time-points from *Δt* = 16 ns to 1.7 ms following retinal photo-isomerization.^24^ We further illustrate how these tools can be used to analyze a sequence of refined crystallographic structures and correlate these with the experimental electron density changes. Finally, the application of these tools to a sequence of difference electron density maps recorded for sub-picosecond time-delays^25^ yields new insight into the time-dependent evolution of an ultrafast structural perturbation (a protein-quake^33,35^) in bacteriorhodopsin.

## METHODS

Difference Fourier electron density maps were calculated as previously described^24,25^ from TR-SFX data recorded from microcrystals of bR at an XFEL. TR-SFX data for the time-points *Δt* = 16 ns, 40 ns, 110 ns, 290 ns, 760 ns, 2 *μ*s, 5.25 *μ*s, 13.8 *μ*s, 36.2 *μ*s, 95.2 *μ*s, 250 *μ*s, 657 *μ*s, and 1.725 ms were recorded at SACLA.^24,40^ TR-SFX data for the overlapping time bins *Δt* = 0 to 141 fs, 49 to 193 fs, 94 to 245 fs, 141 to 314 fs, 193 to 406 fs, 245 to 458 fs, 314 to 490 fs, 406 to 518 fs, 457 to 547 fs, 490 to 574 fs, 518 to 606 fs, 547 to 646 fs, 574 to 695 fs, 606 to 759 fs, 646 to 847 fs, 695 to 946 fs, 759 to 1025 fs, and 847 to 1201 fs, as well as the time-point *Δt* = 10 ps, were calculated from data recorded at beamline Coherent X-ray Imaging (CXI)^25,41^ of the Linac Coherent Light Source (LCLS).

**FIG. 1.**
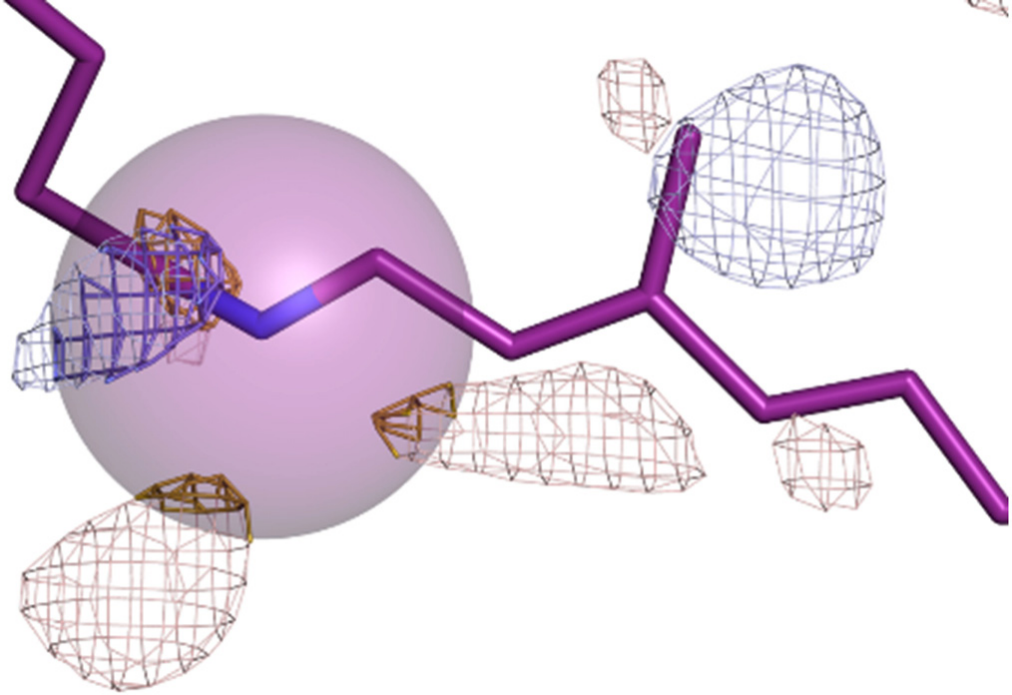
Illustration of how electron density changes are quantified about the atoms contained within a protein structure. A purple sphere 2.0 Å in radius is centered on the protonated Schiff base nitrogen (Nε of Lys216). All difference electron densities within the sphere are selected (yellow represents negative difference electron density; blue represents positive difference electron density, contoured at 3.5 σ). All positive difference density and negative difference density values within this sphere (thick chicken-wire) are averaged independently to obtain the dual-valued amplitude function *A*(*a_i_*), which has both a positive and a negative value for each atom *a_i._* This map corresponds to the time delay *Δt* = 40 ns.^24^

**FIG. 2.**
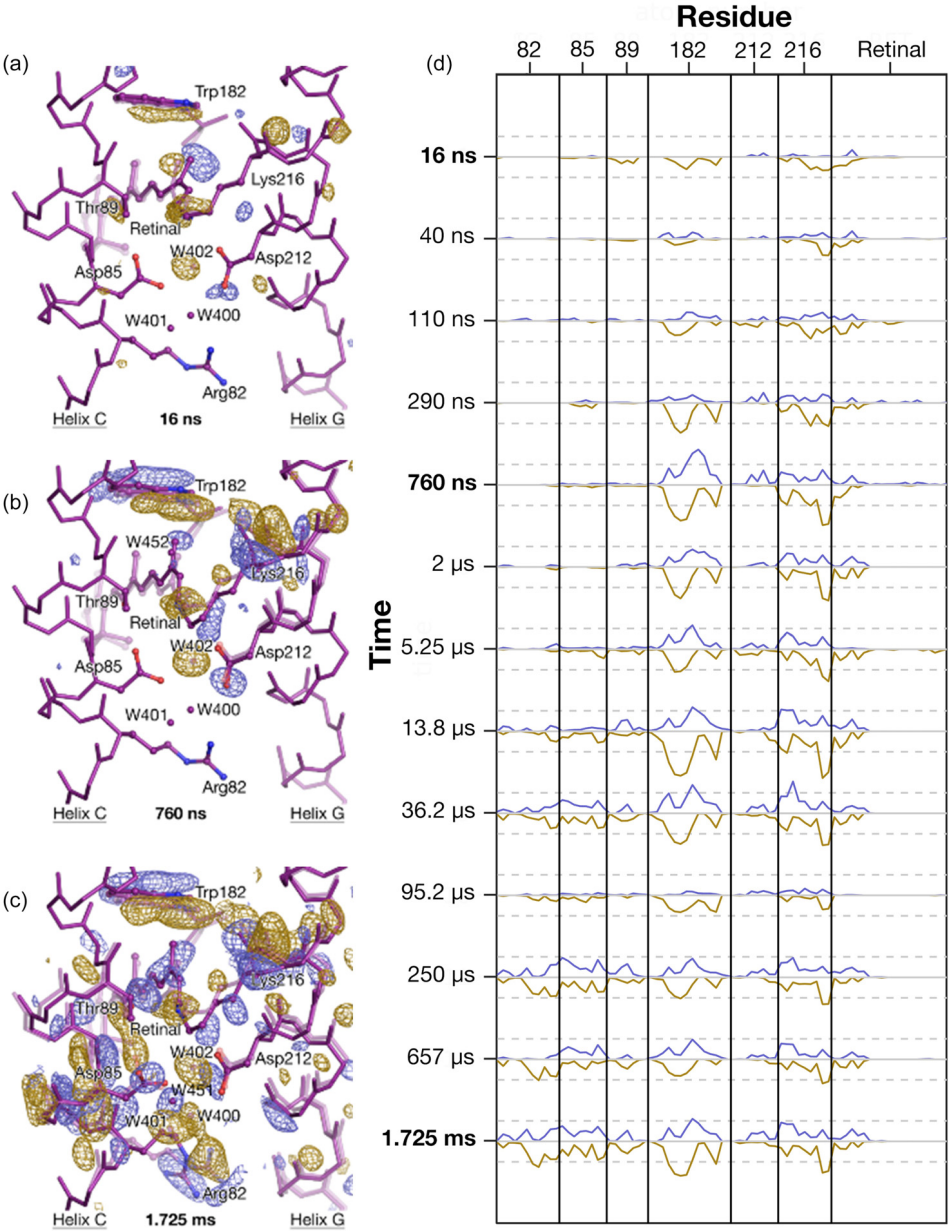
Representation of positive and negative difference density amplitudes as one-dimensional functions. (a)–(c) Selected difference Fourier electron density maps calculated from TR-SFX data for the time points *Δt* = 16 ns (a), 760 ns (b), 1.725 ms (c).^24^ These maps are contoured at ±3.5 σ (yellow, negative difference electron density; blue, positive difference electron density). (d) Difference amplitudes, *A_j_*(*a_i_*), averaged over spheres of radius 2.0 Å about atoms *a_i_* within the protein; electron difference density amplitudes of magnitude ≤3σ were set to 0 before averaging. The atoms of the retinal, Lys216 and Asp212 (helix G), Trp182 (helix F), Arg82, Asp85 and Thr89 (helix C) are chosen in order to represent this analysis. Electron density changes initially associated with the retinal, Lys216 and Trp182 spread with time to other residues of the protein including helix C. Dashed lines represent the value of ±1σ, where σ is the root mean square difference electron density averaged over the unit cell.

**FIG. 3.**
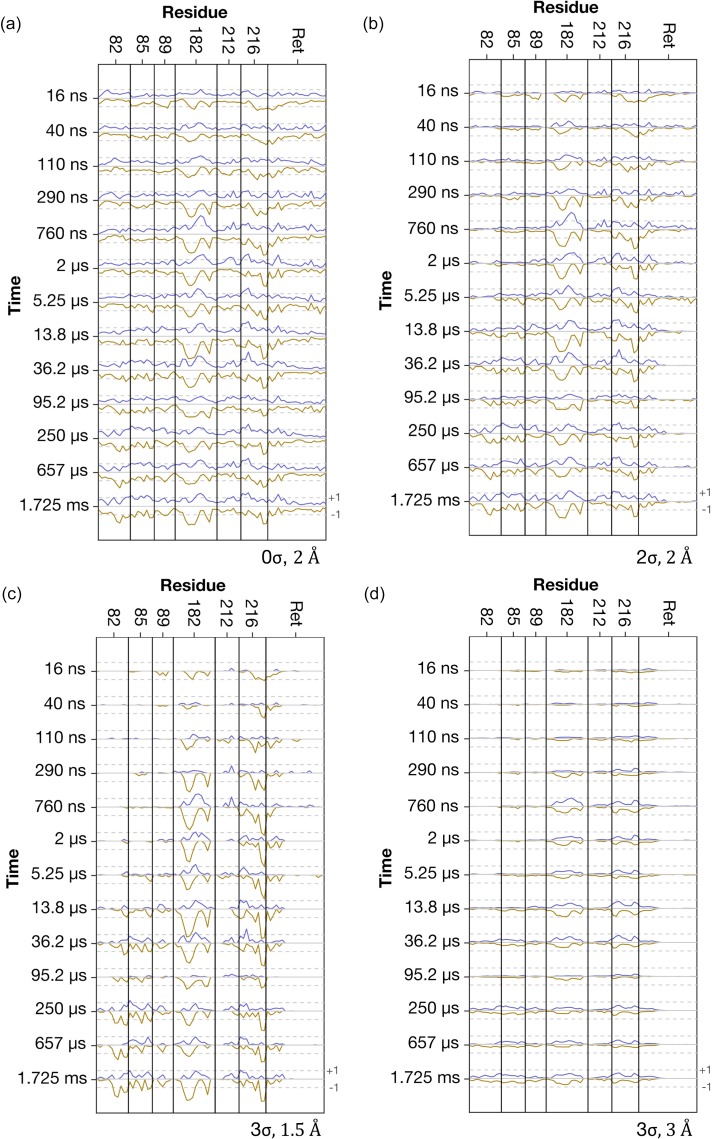
The effect of changing the radius of the sphere of integration or the low-σ floor on the difference amplitudes *A_j_*(*a_i_*). (a) The same data as shown in Fig. 2(d) but with *A_j_*(*a_i_*) calculated with no low-σ floor and a radius of integration of 2 Å. (b) The same data as shown in Fig. 2(d) but with *A_j_*(*a_i_*) calculated with the low-σ floor chosen as 2σ and a radius of integration of 2 Å. (c) The same data as shown in Fig. 2(d) with *A_j_*(*a_i_*) calculated with the low-σ floor chosen as 3σ but with the radius of integration chosen as 1.5 Å. (d) The same data as shown in Fig. 2(d) with *A_j_*(*a_i_*) calculated with the low-σ floor chosen as 3σ but with the radius of integration chosen as 3.0 Å. A lower threshold value of σ increases the baseline values and decreases the sharpness of *A_j_*(*a_i_*). Positive difference density features associated with *A_j_*(*a_i_*) become lost if the radius of integration is too low, whereas *A_j_*(*a_i_*) loses sharpness if the sphere of integration is too large since the spatial overlap of two spheres of integration from one atom to the next increases. Dashed lines represent the value of ±1σ.

**FIG. 4.**
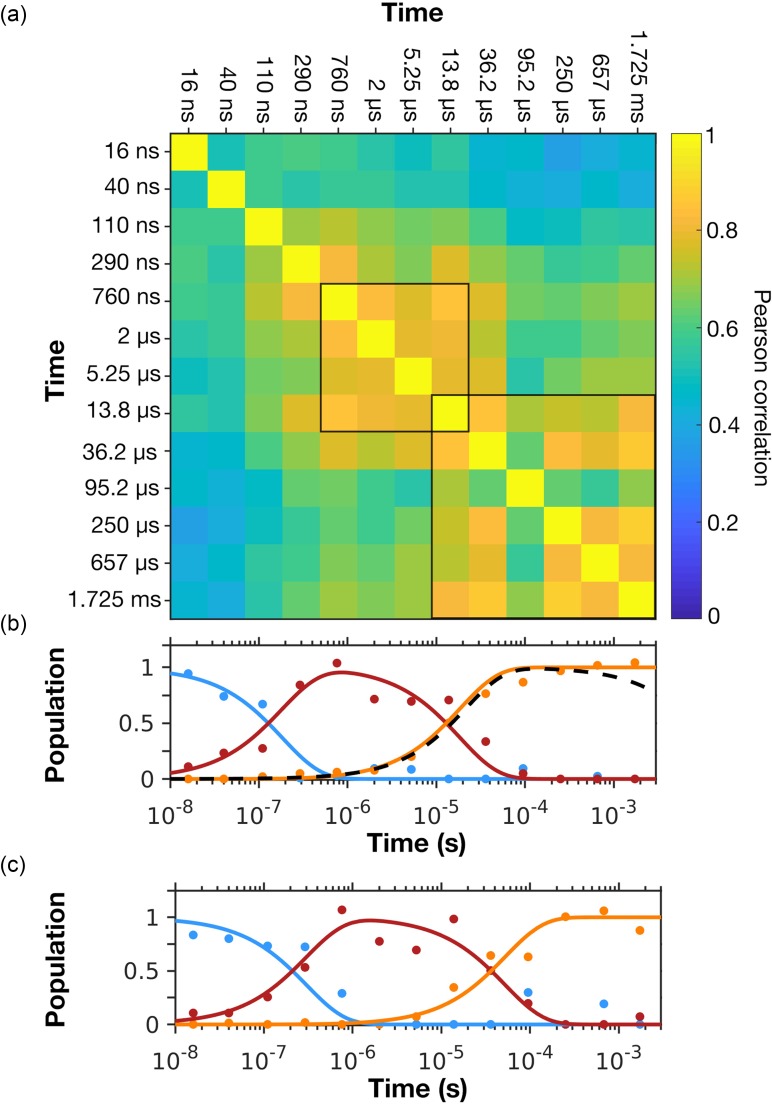
The evolution of correlated structural changes in bR. (a) Pearson correlation values, ρ(*A_j_*(***a***),*A_k_*(***a***)), calculated between the difference electron density amplitudes, *A_j_*(***a***), where *j* corresponds to each of the time-delays *Δt* = 16 ns, 40 ns, 110 ns, 290 ns, 760 ns, 2 *μ*s, 5.25 *μ*s, 13.8 *μ*s, 36.2 *μ*s, 95.2 *μ*s, 250 *μ*s, 657 *μ*s and 1.725 ms. Highly correlated sub-domains for which the average value of the Pearson correlation function ρ(*A_j_*(*a_i_*),*A_k_*(*a_i_*)) ≥ 0.85 across the sub-domain, are bounded by black borders. The time point *Δt* = 95.2 *μ*s is poorly correlated with all other time-points due to the high level of noise in this difference Fourier electron density map.^24^ (b) Results from linear decomposition using three time-dependent components of selected difference Fourier minima and maxima as previously described.^24^ Solid lines are the predicted populations of the three components (first component amplitude blue; second component amplitude red; third component amplitude orange) whereas the dots correspond to the populations resulting from an optimized linear sum of the three components against the experimental data. The dashed black curve indicates the L-to-M spectral transition as measured by time-resolved spectroscopy.^24^ (c) Results from linear decomposition using the time-dependent difference electron density amplitudes *A_j_*(***a***) for the entire protein. A correlation is apparent between the growth and decay of the populations of these three components and the sub-domains within which ρ(*A_j_*(***a***),*A_k_*(***a***)) are strongly correlated.

**FIG. 5.**
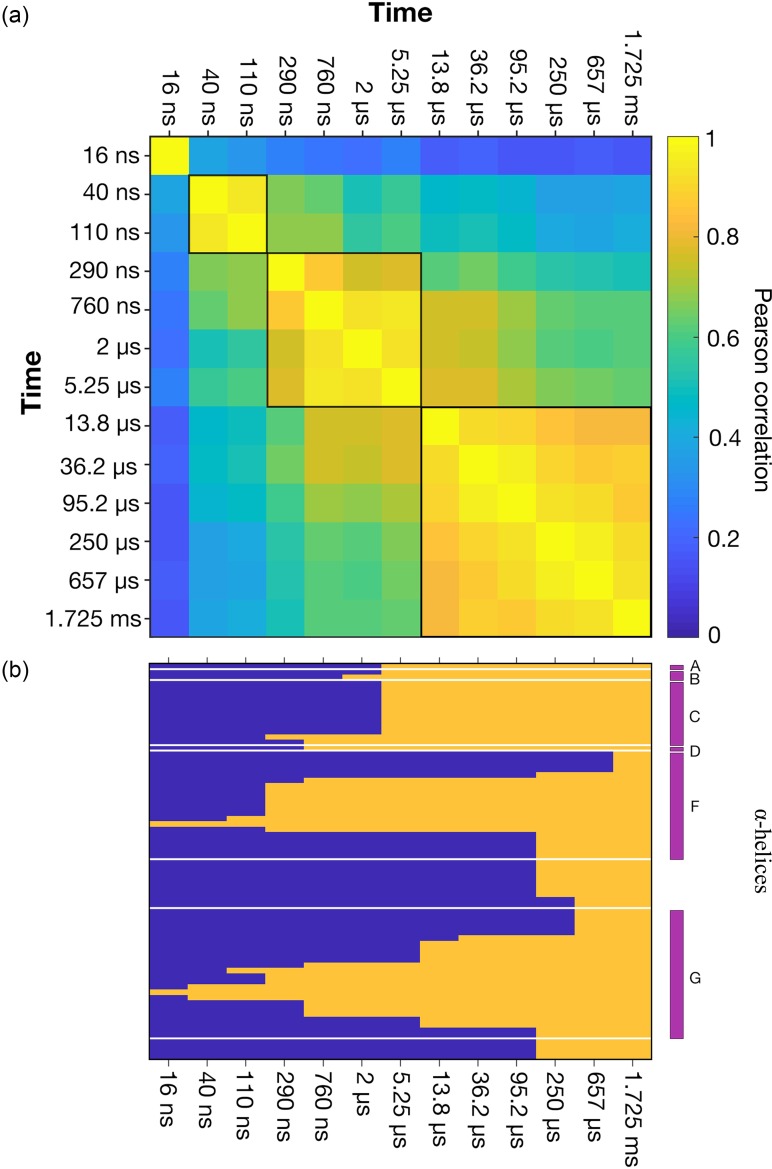
Pearson correlation analysis for the results of structural refinement against TR-SFX data. (a) Pearson correlations values, ρ(*A_j_*(*a_i_*),*A_k_*(*a_i_*)), determined between theoretical difference amplitudes, *A_j_*(***a***), calculated from the deposited crystallographic structures for the time-delays: *Δt* = 16 ns, 40 ns, 110 ns, 290 ns, 760 ns, 2 *μ*s, 5.25 *μ*s, 13.8 *μ*s, 36.2 *μ*s, 95.2 *μ*s, 250 *μ*s, 657 *μ*s, and 1.725 ms. Highly correlated sub-domains for which the average value of ρ(*A_j_*(*a_i_*),*A_k_*(*a_i_*)) ≥ 0.9 are bounded by black borders. (b) Plot of the residues which were allowed to move during structural refinement.^24^ Yellow regions were allowed to move whereas blue regions were held fixed. Regions which did not move for any time-point are not shown. Boundaries to TM-helices are shown. The apparent boundaries between highly correlated regions in the Pearson plot (a) are weakly correlated with when specific residues were allowed to move during structural refinement.

**FIG. 6.**
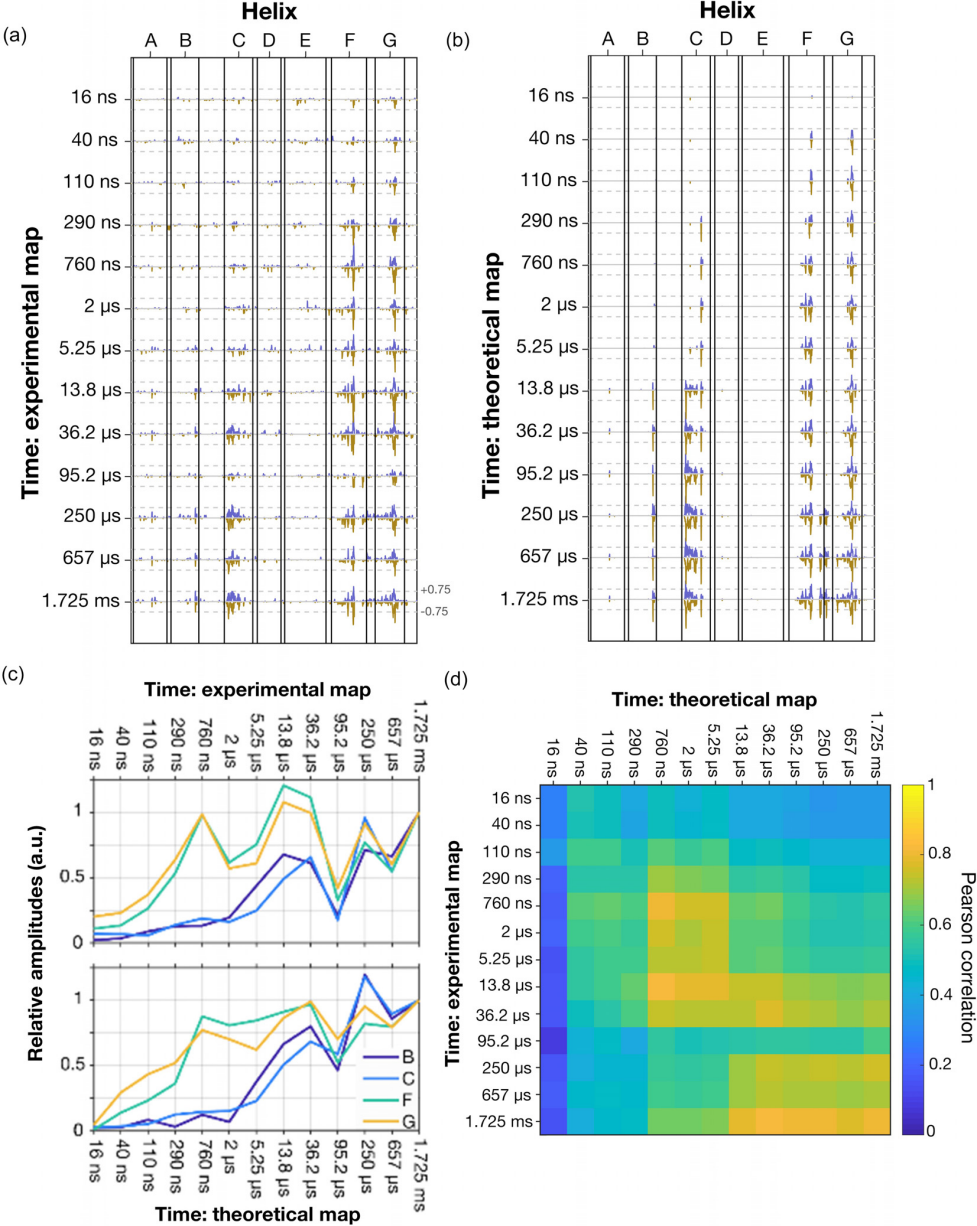
Comparison of experimental difference Fourier electron density maps and those calculated from refined crystallographic structures. (a) Difference amplitudes, *A_j_*(*a_i_*), extracted from a 2.0 Å radius sphere about atoms *a_i_* from the experimental difference Fourier electron density maps. Dashed lines represent the value of ±0.75 σ. (b) Difference amplitudes extracted from theoretical difference Fourier electron density maps calculated from deposited crystallographic structures. In both cases, *j* corresponds to the time-points *Δt* = 16 ns, 40 ns, 110 ns, 290 ns, 760 ns, 2 *μ*s, 5.25 *μ*s, 13.8 *μ*s, 36.2 *μ*s, 95.2 *μ*s, 250 *μ*s, 657 *μ*s, and 1.725 ms.^24^ The y-axis is scaled to facilitate comparison with panel (a). (c) Comparison of the time-evolution of the absolute values of the amplitudes |*A_j_*(*a_i_*)| averaged over regions selected from (a) and (b) for helices B (Met56-Leu58, dark blue), helix C (Ala81-Leu94, light blue), helix F (Leu174-Ser183, green), and helix G (Asp212-Val217, orange) calculated from experimental (top) and theoretical (bottom) difference electron density maps. These amplitudes were normalized to unity for *Δt* = 1.725 ms. (d) Pearson correlations values, ρ(*A_j_*(*a_i_*),*A_k_*(*a_i_*)), determined between experimental difference Fourier electron density maps and theoretical difference Fourier electron density maps for the time-sequence from *Δt* = 16 ns to 1.725 ms. There is no auto-correlation in this calculation, and therefore, this matrix is not diagonal. The slightly diagonal nature of the matrix indicates correlations between experimental and theoretical difference Fourier electron density maps.

**FIG. 7.**
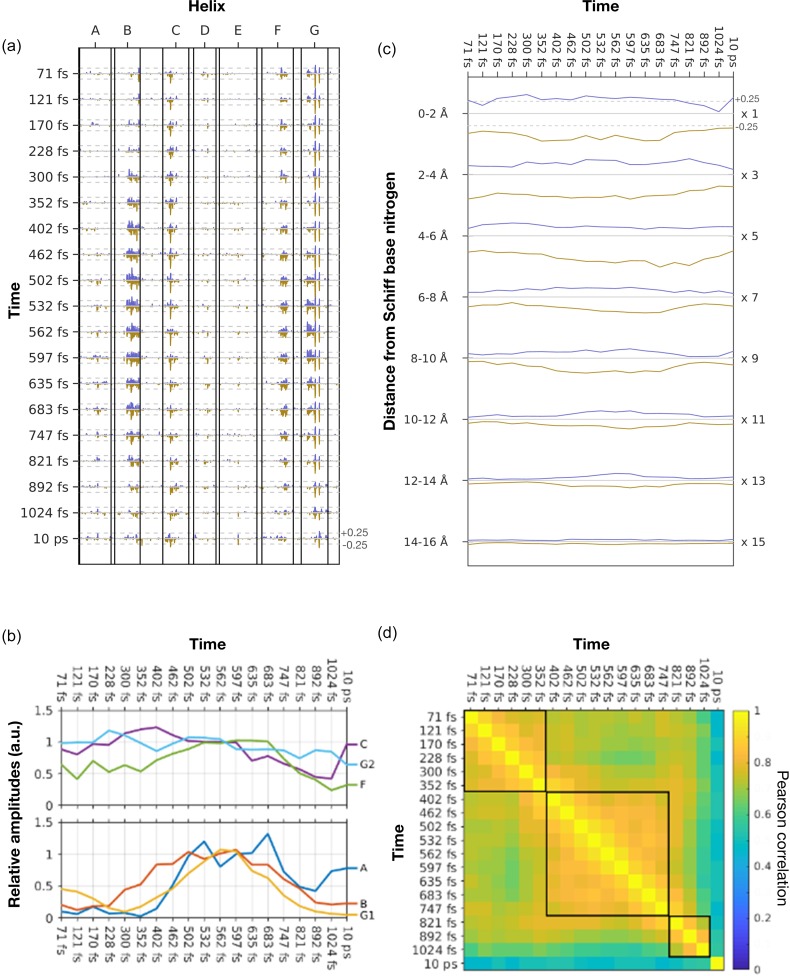
Correlated motions in bR on the femtosecond timescale. (a) Difference amplitudes, *A_j_*(*a_i_*), averaged over difference Fourier electron density maps within a spheres of radius 2.0 Å centered upon atom *a_i_*. Each time-point *j* represents data binned from the time-window *Δt* = 0–141 fs; 49–193 fs; 94–245 fs; 141–314 fs; 193–406 fs; 245–458 fs; 314–490 fs; 406–518 fs; 457–547 fs; 490–474 fs; 518–606 fs; 547–646 fs; 574–695 fs; 606–759 fs; 646–847 fs; 695–946 fs; 759–1025 fs; 847–1201 fs; and 10 ps.^25^ Dashed lines represent the value of ±0.25 σ. (b) Time-dependent amplitudes averaged over the regions of helices A (residues Leu19-Gly21, dark blue), B (residues Thr47-Leu58, red), C (Tyr83-Trp86, purple), F (Trp182-Pro186, green), and G (Asp212, Val213 and Lys216, light blue; and Thr205-Met209, orange). The complex nature of these time-dependent motions is illustrated by the different timescales of movements in different regions of bR. Curves are normalized over the average of 532 fs to 597 fs. (c) Time-dependence of the average values of *A_j_*(*a_i_*) within concentric spherical shells centered upon the Schiff base nitrogen. Values on the y-axis indicate the inner and outer radius of each shell. The average values for *A_j_*(*a_i_*) are scaled by 1× (0 to 2 Å), 3× (2 Å to 4 Å), 5× (4 Å to 6 Å), 7× (6 Å to 8 Å), 9× (8 Å to 10 Å), 11× (10 Å to 12 Å), and 13× (12 Å to 14 Å) to assist with reading these plots. A growth and decay of these average amplitudes are seen within shells more than 6 Å removed from the Schiff base nitrogen. Dashed lines in the uppermost plot represent the value of ±0.25 σ. The scale associated with each of the lower plots should be divided by the scale-factors above. (d) Pearson correlations values, ρ(*A_j_*(*a_i_*),*A_k_*(*a_i_*)), calculated between the difference amplitudes, *A_j_*(*a_i_*). Highly correlated sub-domains for which the average value of ρ(*A_j_*(*a_i_*),*A_k_*(*a_i_*)) ≥ 0.9 are highlighted with black boundaries. Median time-delays within each bin are indicated for each of these bins.

Programs from the CCP4 suite^42^ were used to convert difference electron density maps into Cartesian coordinates through the following steps: (i) preprocessing with mapmask for extending the maps to cover the full cell volume; (ii) conversion of maps to Cartesian coordinates with maprot while decreasing the grid distance to 0.25 Å; (iii) reading the final Cartesian difference electron density maps with map_reader for translation with a Python script into a 3D matrix in h5 format. Electron density amplitudes within each difference electron density map were then analyzed around the atomic position of the atoms *i* of the resting state model using MATLAB.^43^ Spheres of a user specified radius (chosen as 2 Å for Figs. 1, 2, and 4–7, but varied in Fig. 3) were extracted about the selected atom's coordinate and electron density amplitudes were interpolated onto a grid with a mesh spacing of 0.5 Å and with the origin centered upon the chosen atom. A user specified threshold was then applied to set difference electron density values with an absolute amplitude below a certain value to zero. In this work, the floor threshold was chosen as ±3 σ in Figs. 1, 2, and 4–7 which present experimental data, where σ is the root mean square electron density of the cell. The average positive and the average negative density were then calculated separately to yield the (dual valued) amplitude function *A*(*a_i_*) for each atom *i*. The resulting one-dimensional representation of the difference Fourier electron density map, *A*(*a_i_*), could then be analyzed further in MATLAB. These operations included the pairwise calculation of Pearson correlations, the averaging of difference electron density amplitudes over selected regions of the protein, linear decomposition of a sequence of time-dependent difference density amplitude functions *A*(*a_i_*), and the subtraction of difference electron density amplitudes using different radii of integration so as to represent the propagation of electron density changes through concentric spherical shells. The values of *A*(*a_i_*) were dependent upon the choice of the sphere radius and whether or not to impose a low-σ electron density cut-off. The effects of varying the low-σ floor or varying the user specified sphere radius are shown in Fig. 3.

Theoretical difference Fourier electron density maps were calculated from the resting state coordinates and the photo-activated state coordinates. Since the deposited pdb files contained partial occupancy for the photo-activated conformation, these files were rewritten to full occupancy pdb files using the B conformer, which corresponds to the transient photo-activated structure, and with the A conformer (resting state) being removed. Structure factors were calculated with sfall^42^ and the difference maps were calculated using fft.^42^ Four dummy water molecules were put in the excited structural model but outside of the protein, and were not put in the resting model, to allow theoretical difference electron density maps to be scaled relative to each other according to the density amplitudes predicted for these dummy water molecules. For the analysis of theoretical maps (Figs. 5 and 6), the sigma floor was chosen by a comparison of theoretical and experimental *A*(*a_i_*) curves since σ adopted a very different physical interpretation (which depended upon how many residues were moving) in these noise-free theoretical maps.

## RESULTS

### Representation of electron density changes

Our key concept is to translate the information content of a three-dimensional difference Fourier electron density map, FT[(F_obs_^light^ − F_obs_^dark^)·exp(Φ)], into a one-dimensional representation of difference electron density amplitudes, *A*(*a_i_*), where *a_i_* represents the atoms within the protein's pdb file. Our algorithm achieves this translation by: (i) selecting an atom, *i*, within the protein's resting structure; (ii) selecting all difference electron densities within a sphere of a specified radius about this atom for which the sphere radius (Fig. 1) is a user specified input parameter [we use a radius of 2.0 Å for Figs. 1, 2, and 4–7 in this analysis, and results using other sphere radii are given in Figs. 3(c) and 3(d)]; (iii) removing all difference electron densities within this sphere with an amplitude below a user specified contour level below which its value was set to zero in the experimental maps [we use −3.0 σ < amplitude < 3.0 σ for experimental data presented in Figs. 2 and 4–7, results from the choice of other σ floor values are shown in Figs. 3(a) and 3(b)]; (iv) averaging the remaining positive and remaining negative difference electron density values within this sphere to recover the positive and negative amplitudes *A*(*a_i_*) for each atom *i*; and (v) representing these average values on a one-dimensional plot *A*(*a_i_*) as a function of atom number *i* of a pdb file, and this number is plotted along the *x*-axis.

The results from this procedure are illustrated in Fig. 2. Difference Fourier electron density maps are displayed for the time-points *Δt* = 16 ns, 760 ns, and 1.725 ms following photoexcitation of the retinal by a green ns laser pulse [Figs. 2(a)–2(c)]. Results from the above prescription when applied to the sequence of thirteen time-delays from *Δt* = 16 ns to 1.725 ms are shown in Fig. 2(d), which plots *A*(*a_i_*) for atoms selected from within helix C (Arg82, Asp85, and Thr89), helix F (Trp182), helix G (Asp212 and Lys216), and the retinal. This representation of the experimental data highlights the smooth and coordinated nature of the evolution of electron density changes in these regions due to the smoothly varying population of the constituent states. For example, it is apparent from this representation that electron density changes first emerge on the photo-isomerized retinal near the Schiff base and Lys216, to which the retinal is covalently bound via a protonated Schiff base. Electron density changes rapidly emerge on Trp182, which sits directly to the cytoplasmic side of C20 of the retinal and therefore a steric clash between this side chain and the retinal arises upon isomerization. Changes in electron density associated with Trp182, however, plateau from *Δt* = 290 ns. By contrast, the growth of electron density changes along the extracellular side of helix C^44,45^ is at first associated with Thr89 and Asp85 and only at later times do structural changes spread to Arg82.

Different choices of sphere radius and the low-σ floor influence the details of *A*(*a_i_*) and are illustrated in Fig. 3. If the low-σ floor is zero or low, then additional noise arises and increases in the baseline of *A*(*a_i_*) [Figs. 3(a) and 3(b)]. In the extreme case of very high-σ floor, then information is lost since most difference density would be rejected. If the radius of integration is too small, then information regarding positive difference electron density changes is lost [Fig. 3(c)]. Conversely, if the radius is too large then the spheres of integration overlap with each other and as a consequence the baseline of *A*(*a_i_*) is raised and contrast is lost [Fig. 3(d)]. We, therefore, consider a sphere radius approximately equal to the resolution of the map and a low-σ floor of 3σ to be pragmatic choices although these parameters may be changed as appropriate.

We previously represented changes in electron density with time as plots of the maximum (or minimum) values of difference electron density features that were identified in Crystallographic Object-Oriented Toolkit (COOT)^46^ and read out manually (e.g., Fig. 3 of Ref. 24 and supplementary material Fig. S2 of Ref. 25) The representation developed here provides a more objective measure of these changes since there is no subjective choice concerning which difference electron density peak corresponds to which residue; this analysis is not affected by the position of any specific difference electron density maxima/minima varying from one map to the next; difference electron density maps are integrated over spheres of chosen radius rather than maxima and minima being read manually; and the selected regions of interest are specified as user input in a MATLAB code, whereas the manual reading of all peak maxima and minima within a difference electron density map is simply not practical. The following analysis provides some examples that illustrate how this tool offers opportunities to represent the time-evolution of structural changes from a sequence of difference electron density maps in a manner that can aid the functional interpretation of protein structural changes. The tool is flexible and may be helpful in other contexts.

### Correlations of electron density changes

A specific challenge when analyzing a sequence of difference Fourier electron density maps is to decide how many components are most appropriate to describe the electron density changes with time over the entire sequence. SVD approaches^8,37^ and real-space clustering algorithms^38^ have been applied in this context, as well as linear decomposition of a matrix of time-dependent difference electron density amplitudes^24^ SVD, however, has the disadvantage that all but the principal difference electron density components are difficult to interpret whereas linear decomposition involves both a user specified choice of the number of linear components and the manual selection of difference electron density peaks to be incorporated into the analysis. Our approach, which maps a three-dimensional difference electron density map to a one-dimensional representation of positive and negative electron density changes, can be adapted to this challenge. As with real-space clustering algorithms,^38^ our underlying reasoning is that similar structural changes must extend over sub-sequences of difference Fourier electron density maps because of the smoothly varying population of the constituent states, and this should be reflected in the degree of correlation between the amplitudes *A_j_*(*a_i_*), where *j* corresponds to each of the time-points *Δt* of the time sequence.

The Pearson correlation function, ρ(*A_j_*(***a***),*A_k_*(***a***)), for all thirteen time-points (***a*** corresponds to all atoms within the protein and cofactors, structured water molecules, etc.) is presented in Fig. 4 as a two-dimensional heat map. In this calculation, all diagonal elements are unity since the Pearson correlation of any function with itself is always unity. What this representation reveals is that some difference Fourier electron density maps correlate more strongly with others and, to a large extent, the closer in time (on a logarithmic scale) two data-points are the more they correlate. This is intuitively obvious because the closer in time two time-points are the more likely they are to arise from similar populations of the same structural intermediate. For example, the sub-set *Δt* = 13.8 *μ*s, 36.2 *μ*s, 250 *μ*s, 657 *μ*s, and 1.725 ms has a strong correlation with the average value of ρ(*A_j_*(***a***),*A_k_*(***a***))** **≥** **0.85 for all Pearson coefficients within this block [Fig. 4(a)]. This block should also be taken to include the time-point *Δt* = 95.2 *μ*s despite it being poorly correlated with the other experimental data due to the relatively high level of noise in this particular experimental difference electron density map^24^ Similarly, the set *Δt* = 760 ns, 2 *μ*s, 5.25 *μ*s, and 13.8 *μ*s also has a high degree of correlation with the average value of ρ(*A_j_*(***a***),*A_k_*(***a***))** **≥** **0.85 across this block [Fig. 4(a)].

There is an apparent correlation between the boundaries of the correlated blocks identified above [Fig. 4(a)] and the time-points identified as transition points between the intermediate conformations previously identified by linear decomposition^24^ of a selected number of difference electron density peak maxima or minima [Fig. 4(b)]. Specifically, the first component identified by linear decomposition was initially fully populated but decayed on a nanosecond timescale with the second component being highly populated between the time-points *Δt* = 290 ns and 13.8 *μ*s, and the transition from the second to the third component near *Δt* = 13.8 *μ*s coincided almost exactly with the time-evolution of the spectral transition from the L-to-M spectral intermediate.^24^ Linear decomposition of the full set of vectors *A_j_*(*a_i_*) also using three components gave similar results for the timescales of transitions between populations [Fig. 4(c)], although the rise of each component appears to be slightly delayed relative to that recovered from our earlier analysis of a matrix of selected maxima or minima for atoms located quite close to the isomerized retinal [Fig. 4(b)], possibly because difference density features further removed from the active site evolve slightly slower.

Our representation of difference density amplitudes across a sequence of difference Fourier electron density maps, therefore, provides rapid feedback as to the timescale and nature of structural changes throughout the protein without any modeling of these data. We suggest that by first calculating *A_j_*(***a***) for a sequence of difference Fourier electron density maps *j* and then representing the Pearson correlation function between these functions ρ(*A_j_*(***a***),*A_k_*(***a***)), a first assessment as to the nature and number of components appropriate to describe the full experimental dataset can be gained from the number and the size of strongly correlated sub-blocks within this plot [Fig. 4(a)]. This analysis also provides useful feedback as to the quality of the experimental difference electron density data as a whole. Moreover, should SVD analysis or linear decomposition be applied to these experimental maps, then the number of intermediates and the timescales of their rise and decay can be judged against the information represented in a heat map of the function ρ(*A_j_*(***a***),*A_k_*(***a***)) [Fig. 4(a)].

### Correlations between refined crystallographic structures

Another appealing aspect of working with the difference electron density amplitude functions, *A_j_*(*a_i_*), is that it is straightforward to extend this approach to analyze a sequence of refined crystallographic structures. To represent this extension of the method, we again use x-ray crystallographic structures deposited for thirteen time points of bR from *Δt* = 16 ns to 1.725 ms.^24^ The associated pdb files: 5B6W, 5H2H, 5H2I, 5H2J, 5B6X, 5H2K, 5H2L, 5H2M, 5B6Y, 5H2N, 5H2O, 5H2P, and 5B6Z, contain dual conformations with varying crystallographic occupancies of the photo-activated species (16%, 13%, 16%, 24%, 24%, 19%, 21%, 30%, 28%, 18%, 26%, 20%, and 34%, respectively). During structural refinement, the dark conformation of bR (pdb entry 5B6V) was held fixed as both the occupancy and the structure of the photo-activated conformation varied.^24^ We extracted the photo-activated conformation from each of these pdb files, calculated theoretical intensities from the pdb coordinates of the photo-activated and dark conformations, and from this set of intensities, we calculated theoretical difference Fourier electron density maps. From these maps, difference density amplitudes *A_j_*(***a***) and the Pearson correlation values between the functions, ρ(*A_j_*(***a***),*A_k_*(***a***)), were determined in the same manner as when working with experimental data. The results of this analysis are represented in Fig. 5(a).

As may be expected, the absence of experimental noise in these theoretical difference Fourier electron density maps resulted in the contrast between the density amplitude functions *A_j_*(***a***) being larger than when working with experimental data. For example, the Pearson correlation dropped below 40% when comparing the initial structural models with the later structural models [Fig. 5(a)]. The refined crystallographic structures are strongly correlated within three blocks of time-points: *Δt* = 40 and 110 ns, *Δt* = 290 ns to 5.25 *μ*s, and *Δt* = 13.8 *μ*s to 1.725 ms [Fig. 5(a)], which were all selected on the basis that the average Pearson score over each of these blocks ρ(*A_j_*(***a***),*A_k_*(***a***))** **≥** **0.90. To some extent, these correlations are enhanced by choices concerning which residues to vary during structural refinement. These residues were selected by manual inspection of the difference Fourier electron density maps and more residues were allowed to move as time progressed [Fig. 5(b)]. Nevertheless, there is no hard correspondence between the residues which were allowed to vary and the apparent boundaries between the strongly correlated sub-domains. We, therefore, believe that these correlations arise because structural refinement has accurately captured the evolution of structural changes within the experimental data.

### Comparison between experimental and theoretical difference electron density maps

Another demonstration of the toolbox developed here is provided by comparing the difference density amplitudes, *A_j_*(*a_i_*), calculated from both experimental data and refined crystallographic structures. Figure 6(a) presents plots of *A_j_*(*a_i_*) calculated from the experimental data for all thirteen time-points from *Δt* = 16 ns to 1.725 ms^24^ whereas Fig. 6(b) represents *A_j_*(*a_i_*) calculated from the corresponding refined crystallographic structures. This representation highlights that major features of the experimental difference electron density maps and their evolution over time are largely captured by the deposited structures. For example, when the absolute amplitudes of positive and negative difference electron density functions, *|A_j_*(*a_i_*)|, are summed over helices B, C, F, and G, they follow very similar evolutions in time [Fig. 6(c)].

Pearson correlation values, ρ(*A_j_*(*a_i_*),*A_k_*(*a_i_*)), calculated between experimental and theoretical difference density amplitudes provide a quantitative basis for this comparison. In contrast with earlier calculations using only experimental data [Fig. 4(a)] or theoretical maps [Fig. 5(a)], there are no auto-correlations in this calculation and therefore, the diagonal components do not equal unity [Fig. 6(d)]. Moreover, the span from minimum to maximum correlation is typically from 0.2 to 0.8, which reflects a significantly lower correlation between experimental data and theoretical models than is observed for internal correlations between experimental or theoretical maps alone. Nevertheless, the approximate diagonal nature of the correlation function [Fig. 6(d)] illustrates how major features of the experimental difference Fourier electron density maps are captured by partial occupancy structural refinement. These apparent correlations between the difference density amplitudes of experimental and theoretical data could be further strengthened by introducing the Pearson correlation function ρ(*A_j_*(***a***),*A_k_*(***a***)) as an energy term that could be minimized during structural refinement. This could be implemented in analogy with the energy term for chemical constraints associated with chemical bond-lengths and angles in standard crystallographic refinement packages.^42^

### Correlated sub-picosecond motions

XFELs have facilitated time-resolved serial crystallography studies on the femtosecond timescale.^18,19,25,39^ These technical advances provide a new window onto timescales where the thermally assisted crossing of energy barriers is no longer the rate-limiting step and barrier-less protein motions may be excited.^47^ Such motions are expected to be closely related to protein normal modes^48^ and for these reasons, ultrafast protein motions are likely to be global in nature. The term protein-quake was coined to express the idea of quake-like motions propagating out from an epicenter buried within a protein from which the energy of an absorbed photon is unleashed.^33^

A sequence of experimental difference Fourier electron density maps were recently reported from a TR-SFX experiment that probed the structural dynamics of bR on a femtosecond timescale following photo-excitation using a femtosecond laser.^25^ Using these experimental data, we apply the prescription developed above to represent the difference Fourier electron density maps as one-dimensional amplitudes, *A_j_*(*a_i_*), and thereby highlight the evolution of ultrafast structural changes throughout bR [Fig. 7(a)]. This representation highlights motions near the middle of helix C (Tyr83-Trp86), helix F (Trp182-Pro186), and helix G (Asp212, Val213 and Lys216) which all arise rapidly after photoexcitation of the retinal of bR [Fig. 7(b)]. Approximately 400 fs later, motions become visible for helix B (Thr47-Leu58). Since helix B is approximately 1 nm from the photo-isomerized bond which connects Lys216 to the retinal, this delayed motion of helix B corresponds to an approximate velocity of the order of 25 Å/ps, which is in agreement with the speed of sound in proteins as estimated from molecular dynamics simulations.^49–51^ Approximately 100 fs later a similar motion is visible for helix A (Leu19-Gly21). Since this region is slightly more distant from the Schiff base than helix B, this delayed response is also consistent with energy being dissipated as protein motions that propagate away from the site of photo-isomerization at the speed of sound. An alternative representation of these data, for which we use this tool to average the electron density changes within concentric shells centered on the Schiff base nitrogen and plot these changes as a function of Δ*t* [Fig. 7(c)], also highlights the propagation of a structural perturbation away from the Schiff base. This plot reveals that it takes approximately 550 fs for a structural perturbation to propagate a distance of about 13 Å [Fig. 7(c)] which also corresponds to a velocity of 25 Å/ps; that structural perturbations 6 Å to 14 Å away from the Schiff base nitrogen grow and decay in approximately 500 fs; and that structural perturbations further removed from the Schiff base display smaller electron density changes when averaged over the spherical shell volume. Even more complex motions are apparent for the extracellular sub-domain of helix G from Thr205 to Met209, which is initially displaced for the time-window 0 ≤ *Δt* ≤ 245 fs, this motion decreases in amplitude for 228 fs ≤ *Δt* ≤ 402 fs and then returns with a larger amplitude for 402 fs ≤ *Δt* ≤ 747 fs before decaying again by 1024 fs [Fig. 7(b)]. This complex oscillatory motion associated with the extracellular sub-domain of helix G has a periodicity of about 600 fs period. Damped oscillations with a period of a few hundred fs have been suggested from time-resolved Raman spectroscopy on other light-sensitive proteins.^52,53^ In this manner, the visualization tools developed here facilitate a greater appreciation of the complex nature of the protein's ultrafast structural response to the photo-isomerization event. Such motions may be critical in guiding the isomerization event toward a high-yield photo-product.^25,54^

Pearson correlation values between the difference density amplitudes of this time-sequence, ρ(*A_j_*(*a_i_*),*A_k_*(*a_i_*)), are shown in Fig. 7(d). At first sight, this representation suggests less noise in the correlations between experimental maps than for the data from 16 ns to 1.725 ms [Fig. 4(a)], but this conclusion must be tempered by appreciating that all maps from the 0 ≤ *Δt* ≤ 1.2 ps domain were calculated from a running average of the experimental data.^25^ Therefore, these binned data partially overlapped with their neighboring time-points. Nevertheless, it is apparent that there are three strongly correlated domains: 0 ≤ *Δt* ≤ 352 fs, and 402 fs ≤ *Δt* ≤ 747 fs and 821 fs ≤ *Δt* ≤ 1024 fs for which the average Pearson correlation values ρ(*A_j_*(*a_i_*), *A_k_*(*a_i_*)) ≥ 0.86 within each of these blocks [Fig. 7(d)]. The final column within this representation corresponds to a single time-point *Δt* = 10 ps. Since this time point has evolved further and was not binned with any other data, it shows a lower correlation with all other data within this set.

When these experimental data were presented,^25^ five structures were refined and deposited in the protein data bank (pdb entries 6G7H, dark state; 6G7I, *Δt* = 49–406 fs; 6G7J, *Δt* = 457–646 fs; 6G7K, *Δt* = 10 ps; and 6G7L *Δt* = 8.33 ms). These time-windows were selected by comparison with timescales reported for spectral intermediates from time-resolved spectroscopy studies.^55^ In contrast with the slower TR-SFX studies from 16 ns ≤ *Δt* ≤ 1.725 ms,^24^ linear decomposition of the amplitudes of selected difference Fourier peaks was unable to extract the number, nature and timescale of motions when applied to the time-sequence 0 ≤ *Δt* ≤ 1.2 ps.^25^ By comparison, the sub-blocks for which the average Pearson correlation values ρ(*A_j_*(*a_i_*),*A_k_*(*a_i_*)) ≥ 0.86 [Fig. 7(d)] suggest that pooling TR-SFX data from the time-windows: 0 ≤ *Δt* ≤ 352 fs; 402 fs ≤ *Δt* ≤ 747 fs; 821 ≤ *Δt* ≤ 1024 fs, and *Δt* = 10 ps provide a good sampling of the early structural dynamics. We should also highlight that the time domains selected to represent the I (49 ≤ *Δt* ≤ 406 fs) and J (457 ≤ *Δt* ≤ 646 fs respectively) correlate well with the first two of these blocks of data selected using this analysis. We, therefore, again argue that this representation provides a useful tool for judging the nature and timescale of protein motions that arise following photo-excitation and the timescales deriving from our analysis appear to correlate well with characterization using time-resolved spectroscopy.^55^

## CONCLUSIONS

Since the first demonstration of TR-SFX at an x-ray laser five years ago,^17^ the field has developed rapidly, and it is clear that the field of time-resolved serial crystallography will continue to grow. While recent technical advances have opened up ultrafast structural studies and other new experimental opportunities, it is also essential to develop tools for aiding the interpretation of the structural information contained within such data. This is particularly important for characterizing ultrafast motions^18,19,25,35^ and protein motions stimulated by electric fields and THz radiation^29,30^ since protein motions recovered under these conditions are likely to be spread throughout the protein and are not easily understood by looking at a few difference electron density features alone. With this goal in mind, we believe that the representation of time-dependent electron density changes described here provides a useful tool for appreciating the nature of more complex protein motions.

The calculation of the Pearson correlations between the difference amplitude functions permits a sequence of electron density changes to be correlated with time in an intuitive manner [Figs. 4(a) and 7(d)]. This facilitates an assessment of the internal consistency of TR-SFX data across a full dataset and an assessment of the self-consistency of the refined crystallographic structures [Fig. 5(a)]. This representation also highlights the timescale of correlated motions within an ultrafast TR-SFX dataset [Fig. 7(d)], and these timescales were in agreement^25^ with timescales identified by time-resolved spectroscopy.^55^ The velocity at which ultrafast structural perturbations propagate could also be correlated with the speed of sound within a protein [Figs. 7(b) and 7(c)]. This correlation tool also allows time-resolved experimental difference density maps and refined crystallographic structures to be compared (Fig. 6) and may thereby guide decision making during structural modeling. Overall, the toolbox developed here for the analysis of protein structural dynamics is flexible and may be tailored to specific questions, thereby enabling motions to be visualized in a manner that is difficult to grasp when inspecting a sequence of difference Fourier electron density maps alone.
